# Acknowledgement to Reviewers of *Animals* in 2013

**DOI:** 10.3390/ani4010059

**Published:** 2014-02-28

**Authors:** Animals Editorial Office

**Affiliations:** MDPI AG, Klybeckstrasse 64, CH-4057, Basel, Switzerland

The editors of *Animals* would like to express their sincere gratitude to the following reviewers for assessing manuscripts in 2013:

Adams, Nigel J.
Aldridge, Brian
Alexandre-Gouabau, Marie-cécile F.
Anagnostopoulos, Georgios
Asahara, Hiroshi
Babinszky, László
Bacon, Heather J.
Balcombe, Jonathan
Balderer, Werner P.
Balls, Michael
Baragiola, Raúl A.
Beck, Alan
Beck, Ben
Bedard, Alfred
Beitz, Donald C.
Bell, Matthew
Bellarby, Jessica
Bencini, Roberta
Berberich, Gabriele
Biagi, Pier Francesco
Bohling, Justin H.
Brown, David
Bruijnis, Marielle
Buijs, Stephanie
Buller, Henry
Burrow, Heather M.
Caroprese, Mariangela
Carta, Gaspare
Ceballos, Gerardo
Cirulli, Francesca
Cloutier, Sylvie
Clucas, Barbara
Collins, Teresa
Constantin, Angela Petruta
Corbee, Ronald Jan
Cordeiro, Lorraine
Cottle, David J.
Crosson, Paul
Cushing, Nancy
Dacre, Ian
Dale, Robert H. I.
Daniels, Chris
Das, Indraneil
Datar, Isha
Davis, Adrian
de Godoy, Maria R. C.
De Rosa, Giuseppe
Deckers, Jan
Deemer, Danielle
Dellinger, Justin A.
Diederich, Claire
Dinuccio, Elio
Dittoni, Serena
Doherty, Michael
Doyle, Rebecca
Dryden, Gordon
Duffin, Jacalyn
Fairchild, Brian
Farges, Thomas
Faucitano, Luigi
Fazio, Esterina
Ferrante, Valentina
Fine, Aubrey
Foley, Patricia L.
Forkman, Björn
Freake, Michael
Freeman, Elizabeth W.
Freestone, Primrose
Freire, Raf
Fuller, Todd K.
Garstang, Michael
Gavinelli, Andrea
Geers, Rony
Gentile, Arcangelo
Giunchi, Dimitri
Glassey, Steve
Goldingay, Ross
Grace, Delia
Grassman, Lon
Griffin, Brenda
Guinebretiere, Maryse
Halas, Veronika
Hansson, Helena
Haskell, Marie
Hass, Christine
Hässig, Michael
Hauck, Ruediger
Haug, Lore
Hayakawa, Masashi
Head, Josephine S.
Heath, Sebastian E.
Heerkens, Jasper
Heffernan, Claire
Hess, Sebastian
Hirsbrunner, Gabriela
Hoagstrom, Christopher
Hofstede, Gert Jan
Hooker, Sascha K.
Horback, Kristina Marie
Hörning, Bernhard
Huettmann, Falk
Humle, Tatyana
Huxley, Jon
Hyman, Jeremy
Imumorin, Ikhide G.
Innes, John
Izmirli, Serdar
Jachowski, David
Jendral, Michelle
Jenni-Eiermann, Susi
Jennings, Maggy
Johnston, Angie
Jones, Carl
Kamogawa, Masashi
Kazdin, Alan E.
Kiess, Aaron
Kilbride, Amy
Kjarnes, Unni
Kley, Jonas
Kroon, Erna G.
Lagerkvist, Carl Johan
Lamothe, Laurence
Langergraber, Kevin
Langford, Fritha
Lavalli, Kari L.
Laven, Richard A.
Leeb, Christine
Leuenberger, Fanny
Linklater, Wayne
Lischka, Stacy
Lombard, Jason
Lucas, Michael
Luzi, Fabio
Macnulty, Daniel
Macrae, Amelia
MacRae, Rod
Mann, Janet
Manning, Louise
Marchewka, Joanna
María Levrino, Gustavo A.
Matsui, Masafumi
Matthews, Sean
McBride, Anne
Mcbride, Sebastian D
Mccarthy, Kyle
McCobb, Emily
Mcinnes, Colin
Mehrkam, Lindsay
Menzies, Paula
Merlot, Elodie
Meyer-Rochow, Victor Benno
Millar, Kate
Molento, Carla Forte Maiolino
Mormede, Pierre
Mozzachiodi, Riccardo
Muchenje, Voster
Mugnai, Cecilia
Müller, Kerstin
Nagao, Toshiyasu
Nagy, Krisztina
Nannoni, Eleonora
Nekaris, Anna
Nentwig, Wolfgang
Nicholas, Phillipa
Nicol, Christine
Niel, Lee
Nordquist, Rebecca E.
Norwood, F. Bailey
O'riain, Justin
Oliver, Mark
Orams, Mark
Organ, John
Otranto, Domenico
Papastamatiou, Yannis
Parsons, Chris
Pasmans, Frank
Patronek, Gary
Patterson, Brent
Peterson, Mandy
Pétillon, Julien
Piccirillo, Alessandra
Preininger, Doris
Quattrocchi, Fedora
Ranieri, Gaetano
Rea, Roy V.
Reefmann, Nadine
Reiner, Gerald
Richeson, Nancy E.
Robins, Dr Andrew
Rogers, Lesley J.
Rostagno, Marcos
Rowan, Andrew
Rushen, Jeff
Sandøe, Peter
Saucier, Linda
Savell, Jeff
Scagliarini, Alessandra
Schlacher, Thomas A.
Scuffham, Andrew
Selle, Peter H.
Shiro, Brian
Shivik, John
Siniscalchi, Marcello
Sønderskov, Kim Mannemar
Sossidou, Eva
Spanier, Ehud
Spengler Neff, Anet
Stafleu, Frans
Stanley, Margaret
Stanton, Amy
Stavisky, Jenny
Stella, Judi
Stephens, Neil
Stoeger, Angela
Stookey, Joseph
Stott, Alistair
Stull, Jason W.
Sufka, Kenneth J.
Tarlton, John F.
Temby, Ian
Thanassoulas, Constantine
Thiriet, Dominique
Tonsor, Glynn
Trevisi, Erminio
Tzouramani, Irene
Ursin, Lars Oystein
Van De Weerd, Heleen
Van Heezik, Yolanda
Vanhonacker, Filiep
Villaroel, Morris
Wagner, Norman
Wakabayashi, Kaori
Wealleans, Alexandra L.
Weiss, Alexander
Wemelsfelder, Francoise
Whitehead, Neil
Whittaker, Alexandra
Williams, Adrian
Wognum, Nel
Zöls, Susanne
Zulch, Helen

